# Functional Capacity of Institutionalized Older People and Their Quality of Life, Depressive Symptoms and Feelings of Loneliness: A Cross-Sectional Study

**DOI:** 10.3390/nursrep14040229

**Published:** 2024-10-23

**Authors:** Fátima Cano, Elisabete Alves, Lara Guedes de Pinho, César Fonseca

**Affiliations:** 1 Local Health Unit of Baixo Alentejo, 7801-849 Beja, Portugal; d52899@alunos.uevora.pt; 2 Saão João de Deus School of Nursing, University of Évora, 7000-811 Évora, Portugal; lmgp@uevora.pt (L.G.d.P.); cfonseca@uevora.pt (C.F.); 3 Comprehensive Health Research Centre (CHRC), 7000-811 Évora, Portugal

**Keywords:** institutionalized older, functionality, loneliness, depression, quality of life

## Abstract

Background: The increasing number of institutionalized older individuals worldwide stresses the need to evaluate the association between the functional profile of institutionalized older adults and their quality of life (QoL), depressive symptoms and feelings of loneliness. Methods: A cross-sectional study was conducted in 19 residential facilities in Alentejo, Portugal. Between March and September 2023, all individuals aged ≥65 years were invited to complete a structured questionnaire (*n* = 1303). Sociodemographic and clinical data were collected, and validated scales for the Portuguese older population were used. Linear regression and unconditional binary logistic models were computed. Results: The highest level of dependence was observed in the self-care dimension (mean (SD) = 2.93 (1.21)), with 40% of participants exhibiting levels of dependence requiring daily care or total replacement. QoL was inversely associated with functionality in all dimensions, as well as with severe or complete dependence, even after adjusting for sex, age and education. Participants with depressive symptoms and feelings of loneliness were, respectively, three and two times more likely to be dependent on care (adjusted OR = 3.69, 95% CI: 1.80–7.52; adjusted OR = 2.04, 95% CI: 1.07–3.87). Conclusions: Public policies and interventions should include social and emotional support strategies alongside traditional medical interventions.

## 1. Introduction

The progressive increase in the older population poses sociological and healthcare challenges, particularly regarding the health policies aimed at people aged 65 years or older [1,2]. In fact, the increasing aging of the population and the increase in average life expectancy are reflected in the higher concern for the care of the older population, especially because of the need to implement long-term care responses [2,3,4]. This scenario of epidemiological and demographic changes, marked by a notable decrease in mortality and a greater control of early mortality, is responsible for the growing number of people of advanced age. At the same time, demographic aging results in opportunities and challenges of different dimensions on a global scale in the economic, social and health domains. It is undoubtedly one of the main public health concerns due to the increase in noncommunicable chronic diseases due to a decline in the ability to provide self-care [5,6,7,8,9].

The longevity process is a worldwide phenomenon marked by specific biopsychosocial changes [10,11,12,13], namely genetic, physiological, behavioral, psychological and social changes [13,14]. It is a natural process of life during which people continue to aspire to maintain their health and quality of life (QoL). In this context, it is not enough to ensure that each person lives longer; it is also necessary to ensure their autonomy, capacity for self-care, independence and QoL. The functional health of older people has been associated with the perception of well-being, socialization and support networks, intellectual conditions, emotional states and attitudes toward individuals and the world [15]. Functional capacity has attracted increasing attention because the loss of functionality increases the number of chronic diseases and compromises the capacity for self-care, often leading to the search for social solutions, namely institutionalization, which requires adaptations by the health and social sectors [16,17,18].

The ability to maintain functional capacity is directly related to an older person’s QoL and to their ability to care for themselves. It is associated with self-esteem, personal well-being, socioeconomic level, health status, emotional stability, social interaction, activity and self-knowledge [19,20,21,22]. According to previous literature, older people living in nursing homes or other residential structures tend to feel lonely and dissatisfied and are removed from their social networks in the monotony of day-to-day life. This feeling is aggravated and explained by the growing increase in cases of depression, compromised self-esteem, the permanent desire to return to the family, aggression and suicide attempts [13,20].

Depression and loneliness are significant concerns among older people, especially among those who are institutionalized. Recent studies have shown that loneliness is strongly associated with increased depressive symptoms in the older population [23], which may compromise the mental health and QoL of these individuals [24,25]. Thus, attention to the emotional and social aspects of older individuals is crucial to promoting their general well-being.

Portugal is facing significant demographic challenges, which are characterized by an aging population and a low birth rate, making it one of the countries with the greatest demographic fragility in the European Union [2,11]. These challenges are especially pronounced in the Alentejo region, an area that exemplifies the country’s broader demographic problems. The combination of low birth rates, high youth emigration rates and a growing number of older people puts significant pressure on social and health services in the region [26]. Moreover, low population density and depopulation exacerbate feelings of loneliness and social isolation, which are factors that negatively impact the health and well-being of older people [1,2,13].

In this context, a better understanding of how functional capacity is related to the mental health and well-being of older people will contribute to the development of more effective strategies for health promotion in vulnerable populations. Nursing, as an innovative profession, is crucial in addressing these challenges by introducing new approaches, technologies, and practices to improve the quality of care, the autonomy of older adults and the efficiency of services [27]. Thus, this study aims to evaluate the associations between the functional profile of institutionalized older people and their QoL, depressive symptoms and feelings of loneliness according to their sociodemographic and clinical characteristics.

## 2. Materials and Methods

This cross-sectional study was approved by the Ethics Committee of the University of Évora (Reference 22064). Each participant signed an informed consent form.

Between March and September 2023, 11 institutions in the district of Beja and 8 in the district of Évora were invited to participate in the study. The institutions were contacted by sending an email with all the information about the study to be performed, and all of them agreed to participate. Each institution selected specific collaborators, all of whom were health professionals (nurses, psychologists, physiotherapists, social workers and occupational therapists) who provided care at the participating institutions, to perform the data collection. To ensure consistent training and the quality of the data collection, all health professionals were invited to participate in face-to-face and online training where the study objectives of the study and all procedures to be implemented were clarified, including the presentation and description of the questionnaires to be applied. The training sessions for the professionals took place between January and March 2023.

Data collection from the participants took place between March and September 2023, encompassing a total of 1522 people residing in Central Alentejo and Baixo Alentejo. The present included only participants aged 65 years or older who were institutionalized in a residential structure for older people and who were able to freely sign the informed consent form or who had a guardian or a legally recognized significant other who could do so. Thus, 1396 participants met the inclusion criteria of this study. All the participants answered a structured questionnaire for the collection of demographic and clinical data and for the evaluation of the functionality profile (Elderly Nursing Core Set) and cognitive function of the participants (Mini Mental State Examination). A total of 93 participants who did not respond to the functionality questionnaire were excluded from our sample, and our final sample consisted of 1303 institutionalized people aged 65 years or older. Data, namely sex, age, marital status and education, were collected for the sociodemographic characterization of the participants. Clinical data on the medical diagnoses of the participants were also collected. On the basis of these data, multimorbidity was defined as the diagnosis of two or more chronic diseases.

The Elderly Nursing Core Set (ENCS) is an instrument for assessing the functionality of older adults and consists of 31 questions based on the International Classification of Functioning, Disability and Health (ICF), categorized on a 5-point Likert scale. It assesses four dimensions: self-care (washing, dressing, taking care of body parts, moving around using some type of equipment, walking, performing daily routines, maintaining body position, changing basic body positions, care related to the processes of excretion, use of the hand and arm and drinking and eating); learning and mental functions (emotional functions, orientation functions, attention functions, memory functions, consciousness functions and higher-level cognitive functions); communication (speaking, conversation, communicating and receiving oral messages and family relationships); and relationships with friends and caregivers (personal care providers and personal assistants, health professionals and friends) [11]. The ENCS has high reliability (Cronbach’s alpha = 0.963), and there is a strong correlation between items (KMO = 0.947). A higher score indicates a worse functional profile [28,29], with the scores categorized as follows: no problem: 0–4%; mild problem: 5–24%; moderate problem: 25–49%; severe problem: 50–95%; complete problem: 96–100%.

The Mini Mental State Examination (MMSE) assesses cognitive function in six areas: orientation, temporal and spatial, short-term memory (immediate or attention) and recall, calculation, movement coordination and language and visuospatial skills [30]. It is adapted for Portugal, with a score that varies between 0 and 30 [31]. The criterion for the definition of cognitive impairment varies according to the level of education of the participants: a score ≤ 15 for individuals with no formal education; a score ≤ 22 for individuals with <12 years of education; and a score ≤ 27 for individuals with 12 years of education or more [31].

All participants who did not exhibit cognitive impairment (*n* = 447) also completed questionnaires validated for the Portuguese population and adapted for older people to assess QoL (World Health Organization Quality of Life—BREF (WHOQOL-BREF)), depressive symptoms (Patient Health Questionnaire 9—PHQ-9), and feelings of loneliness (Loneliness Scale for Portuguese Elderly—UCLA).

The World Health Organization Quality of Life–BREF (WHOQOL-BREF) measures the QoL of adults in four domains: physical, psychological, social relationships and the environment [32]. The WHOQOL-BREF scale ranges from 0 to 100, with higher scores representing a better QoL. Usable for various disorders and healthy individuals, it allows the calculation of a global indicator of QoL [33].

The 9-item Patient Health Questionnaire (PHQ-9) assesses depressive symptoms via nine questions, each with four response options that reflect the frequency of symptoms, ranging from 0 (none) to 3 (almost every day). This instrument can be used to systematically identify symptoms of depression, and the scores range from 0 to 27: 0–4 points, no depression; 5–9 points, mild depressive disorder; 10–14 points, moderate depressive disorder; 15–19 points, moderately severe depressive disorder; and 20–27 points, severe depressive disorder. The higher the score is, the more severe the depressive symptoms are [34]. It has been validated for the Portuguese population, with good internal consistency and convergent validity [34,35].

The Loneliness Scale for Portuguese Elderly (UCLA) assesses feelings of loneliness with 16 items, which are divided into social isolation and affinities, with responses at four levels. The score ranges from 16 to 64, with scores above 32 indicating significant loneliness [36]. This scale has temporal stability (α (16 items) = 0.985), internal consistency (0.905) and factorial validity, demonstrating high reliability as a diagnostic tool for geriatric loneliness [37].

Data processing was performed via the statistical program STATA 15.1 (College Station, TX, USA, 2017). The sample characteristics are presented as counts and proportions or means and standard deviations. Linear regression models were used to evaluate the crude and adjusted associations and their respective 95% confidence intervals (95% CIs) between the sociodemographic and clinical characteristics of the participants, QoL, depressive symptoms and loneliness with each of the functioning subscales. Unconditional binary logistic regression models were fitted to calculate crude and adjusted odds ratios (ORs) and their respective 95% CIs to assess the determinants of severe or complete functioning.

## 3. Results

The participants had a mean age of 86 years; more than 70% were female, and 16.7% were married or in a common-law relationship (Table 1). With respect to education, 43.4% had not attended school, whereas 54.1% had attended school but not higher education. The physical health of the participants was marked by a high prevalence of multimorbidity (84.1%) and cognitive impairment (64.7%). Among the participants without cognitive impairment, the mean value (SD) of QoL in general was 52.3 (19.2). The mean (SD) QoL ranged from 53.3 (11.2) for the physical domain to 63.8 (13.7) for the environmental domain. The mean score (SD) for depressive symptoms was 5.1 (5.0), and that for feelings of loneliness was 29.5 (10.2).

**Figure 1:**
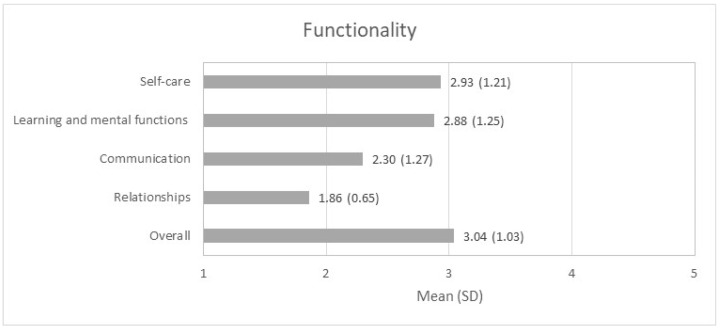
Functional profile of the participants (*n* = 1303).

The distribution of the results of the functional profile of the sample are shown in Figure 1. Specifically, the highest levels of dependence were observed in the dimensions of self-care (mean (SD) = 2.93 (1.21)) and learning and mental functions (mean (SD) = 2.88 (1.25)). Conversely, the relationships dimension appeared to have the lowest levels of dependence and, consequently, better functionality (mean (SD) = 1.86 (0.65)).

**Figure 2:**
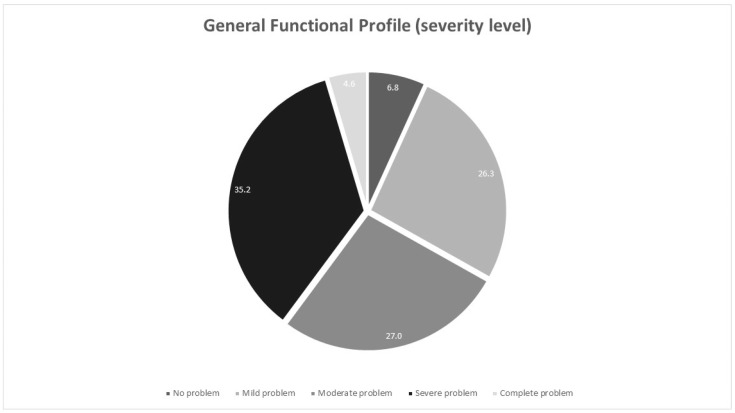
General functional profile of the participants (*n* = 1303).

The mean (SD) value of the general functional profile (3.04 (1.03)) indicates that more than half of the participants needed help with care. In fact, 27.0% of the participants had moderate levels of dependence, while almost 40% reported high levels of dependence that required daily care (severe problem) or total replacement (complete problem) (Figure 2).

Table 2 shows the associations between the sociodemographic, clinical and cognitive characteristics of the participants and their functionality according to the different dimensions of the ENCS. Women, older participants and those with cognitive deterioration tended to obtain significantly higher scores on the functionality scale, indicating higher levels of dependence in all dimensions. Conversely, participants who had attended school tended to have lower scores than those who did not attend school, indicating a higher level of functionality (general functionality: β = −0.45, 95% CI −0.56 to −0.34; self-care (β = −0.44, 95% CI: −0.57 to −0.31), learning and mental functions (β = −0.57, 95% CI: −0.70 to −0.44), communication (β = −0.48, 95% CI: −0.62 to −0.35) and relationships (β = −0.26, 95% CI: −0.33 to −0.18). Marital status did not seem to be significantly associated with the functionality of the participants.

**Figure 3:**
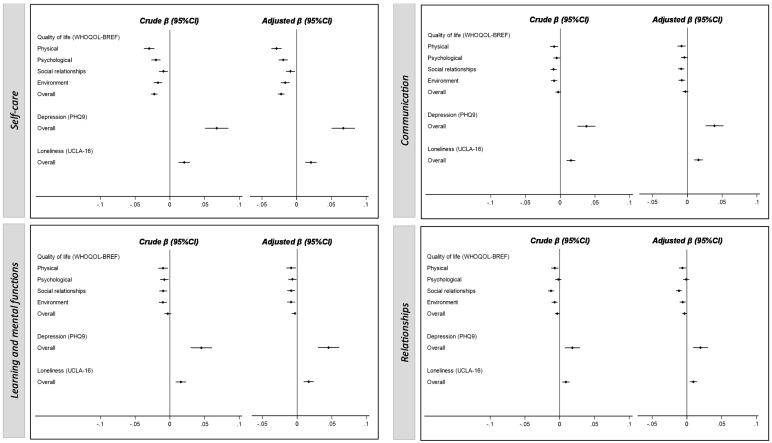
Crude and adjusted associations of the functionality domains with quality of life, depressive symptomatology and loneliness among institutionalized older adults. Crude β, unstandardized beta; 95% CI, 95% confidence interval; adjusted for sex, age and education level.

Figure 3 depicts the crude and adjusted associations of each of the functionality domains with quality of life, depressive symptomatology and loneliness among institutionalized older adults. Among the participants without cognitive deterioration, QoL was inversely associated with the functionality score in the four dimensions evaluated, whereas depressive symptoms and feelings of loneliness were directly associated with higher levels of functional dependence. The same patterns were described even after adjustment for sex, age and education (Figure 3). Only in the communication and relationship domains of the functionality score, the psychological dimension of QoL (β = −0.00, 95% CI: −0.01 to 0.00; and β = −0.00, 95% CI: −0.01 to 0.00, respectively) and the general dimension of QoL (β = −0.00, 95% CI: −0.01 to 0.00; and β = −0.00, 95% CI: −0.01 to 0.00, respectively) did the associations not reach significance. However, the same association trend was described, despite the lack of statistical significance.

Table 3 shows the sociodemographic, clinical, cognitive and psychological characteristics of the participants that were associated with impaired functionality, taking into account the highest level of dependence (severe and complete disorder). Similar to the results of the analysis of the functionality scale by domain, women (OR = 1.41; 95% CI 1.10–1.81) had a greater prevalence of impaired functionality than did men, whereas education (OR = 0.46; 95% CI 0.37–0.58) was negatively associated with the functional dependence of older individuals. Adults over 65 years of age, institutionalized and with cognitive impairment were 22 times more likely to be dependent on care (OR = 22.07; 95% CI 14.60–33.36) than those without cognitive impairment. Among participants without cognitive impairment, the presence of moderate to severe depressive symptoms and feelings of loneliness were significantly associated with impaired functionality. Quality of life seemed to be inversely associated with severe or complete dependence; however, the results were significant only for the physical domain (adjusted OR = 0.96 (95% CI: 0.93–0.99), social relationship domain (adjusted OR = 0.96 (95% CI: 0.94–0.99)) and environmental domain (adjusted OR = 0.96 (95% CI: 0.93–0.99), both in the raw analysis and after adjustment.

Participants with depressive symptoms were three times more likely to be dependent on care (adjusted OR = 3.69 (95% CI: 1.80–7.52)), whereas those who felt lonely were twice as likely to need care (severe problem) or total replacement (complete problem) (adjusted OR = 2.04 (95% CI: 1.07–3.87)) after adjustments for sex, age and education level.

## 4. Discussion

The results of this study provide a detailed view of the functionality of older people, revealing important associations with the sex, education and cognitive impairment of the participants. These findings provide valuable insights into the variables that influence functional dependence in different populations. Presentation of the data by the dimension of functioning and degree of severity provides a rich and multifaceted analysis of the functioning of the participants, allowing the identification of specific needs, the evaluation of the global impact, the stratification of risks and the customization of interventions. Moreover, the analysis of data on the functionality, QoL, depressive symptoms and loneliness of institutionalized people aged 65 years or older provides a solid basis for the discussion of the challenges faced by this population. Our results revealed very high levels of functional dependence. Although the literature supports a higher prevalence of functional limitations in older people, it is important to note that the severity observed in our sample exceeds some of the previous estimates [5,12], suggesting the need for more targeted and comprehensive interventions to adequately meet the needs of this population.

Women and those with cognitive impairment tend to obtain significantly higher scores on the functionality scale, indicating higher levels of functional dependence in all the dimensions evaluated. This finding is consistent with the literature, which suggests that aging and cognitive impairment are strongly associated with decreased functional capacity [17], imposing a greater burden on both participants and institutions in regard to providing care and requiring more resources and specialized support [5,6,17]. In addition, women often report higher levels of functional dependence, possibly due to greater longevity, a higher prevalence of chronic conditions than that of men, and differences in the social structure and support available to older women [38,39,40].

The high prevalence of multimorbidity among participants is consistent with recent findings. The literature indicates that multimorbidity is increasingly common among older people, particularly at older ages, affecting approximately 60–80% of the older population [41]. The management of multimorbidity requires a holistic approach that integrates specialized and generalist care to address the multiple needs of patients [42]. The lack of statistical significance in our study between most dimensions of functioning and multimorbidity may be due to the high prevalence of multimorbidity, which makes our population more homogeneous in relation to this variable and makes it difficult to obtain statistically significant results.

Participants who had attended school had significantly lower scores on the functionality scale, indicating greater functional independence. These data suggest that education may be a protective factor against the loss of functionality, possibly due to the constant cognitive stimulation and access to better living conditions that education provides. This result, although in agreement with the literature [43], highlights the existence of social inequalities in health, which affect health throughout the life cycle, stressing the need to intervene early in less-educated populations who are, consequently, more vulnerable to adverse health outcomes.

Among participants without cognitive impairment, better QoL is associated with lower levels of functional dependence, whereas depressive symptoms and feelings of loneliness are directly associated with higher levels of functional dependence. The associations described between quality of life and all domains of the functionality scale are consistent with the literature. This highlights the importance of social interactions and effective communication in maintaining the mental health and well-being of older people [44,45] as well as of their involvement in lifelong learning activities to improve their mental health and QoL [46]. Moreover, depression can decrease motivation and energy, worsening the functional capacity of older individuals [40,47], whereas loneliness can aggravate mental and physical health problems, increasing dependence [47,48]. These findings reinforce the importance of psychological well-being and social support networks in maintaining functionality in individuals without cognitive impairment [17,42,47]. Thus, programs that promote and encourage social interaction and improve communication skills may be beneficial for this population.

This study highlights crucial issues that require interventions and social and emotional support strategies that go beyond traditional medical interventions. The reduced ability of older people to perform daily activities and participate in social activities affects their physical and mental well-being [5,7,20]. Social isolation, a common problem among those whose health conditions limit mobility or cause stigma [17,23], can exacerbate feelings of dependence and loss of autonomy [7,24], leading to an increased psychological and emotional burden [46,47]. Thus, it is essential to implement multidimensional interventions that disseminate programs that promote social engagement, namely through support groups, community groups and recreational activities, to mitigate feelings of loneliness and depression. In addition, access to counseling and therapy services may help older adults cope with depression and the emotional challenges associated with the loss of functionality. In fact, care strategies that combine medical treatment with psychosocial approaches are essential to address the multiple aspects that affect QoL.

One of the main advantages of this study is the deepening of the understanding of the complex interactions among functionality, mental health, loneliness and quality of life in institutionalized older individuals, which should contribute to the design and implementation of integrated approaches that consider both physical and emotional aspects in the well-being of institutionalized older individuals. Also, the use of multiple data collection instruments validated for the Portuguese population and adapted for older people and their ethical and methodological rigor need to be stressed as strengths of the study. Our study presents a relatively large sample size, which was calculated to ensure a power of 80% to demonstrate associations with a magnitude of at least 1.2 (odds ratio) at a 5% significance level. Also, the inclusion of 11 institutions in the district of Beja and 8 in the district of Évora and the small proportion of participants that did not respond to the functionality questionnaire (6.7%) ensures the representativeness of our sample for the Alentejo region.

Some limitations also need to be acknowledged and discussed. The cross-sectional nature of our study prevents the establishment of causal relationships between functionality and quality, depression and loneliness. The data collection by the institution’s health professionals, although promoting performance in a safe context where the older people feel comfortable, may have influenced participants to respond in a socially desirable way to the questions about QoL, depression and loneliness [19]. However, in this case, it would be expected that the associations described would be even more pronounced, emphasizing the importance of intervening in mental health as early as possible to optimize the quality of life of people aged 65 years or older. The proportion of women included in our study was higher than that described for the older population (65 years of age or older) in Portugal [49]. In 2021, women accounted for approximately 57% of the older population, reflecting greater female longevity. The Alentejo region, where the study was conducted, has a proportion of older people above the national average, with Central Alentejo and Baixo Alentejo having one of the highest percentages of older people in the country. Thus, the high average age included may also explain the greater participation of women. This phenomenon has been called the “feminization of old age” in scientific literature [50]. However, although women live longer than men, these additional years are generally not accompanied by good health, as they tend to have poorer health throughout their lives and greater economic needs [2]. In the current study, a sensitivity analysis was performed by randomly selecting a similar number of male and female older adults, and the results remained very similar. Finally, the analysis of QoL, depressive symptoms and loneliness was restricted to participants without cognitive impairment, which reduced the sample size and consequently limited the statistical power. However, this procedure is common in most studies in this area because it is commonly accepted that the self-completion of psychosocial assessment scales requires adequate cognitive ability to ensure the attainment of valid and reliable responses [51]. Thus, the restricted application of the QoL, depression and loneliness scales to individuals without cognitive impairment strengthens the internal validity of the study, ensuring that the analyzed data more accurately reflect the perception of each participant, thus ensuring the quality and reliability of the data obtained.

## 5. Conclusions

The results of this study are in line with the literature and more recent guidelines, which highlight the importance of public policies and interventions focused on education and psychosocial support to improve functionality, QoL and mental health, especially among vulnerable populations [52,53,54,55]. The increase in longevity has profound implications for health policies and social responses, requiring strategies that are adapted to the complex needs of older people. In this context, programs that promote healthy aging and preventive and integrated approaches to chronic diseases, including continuing education and social support, may be effective in reducing functional dependence and contribute to improving the QoL and mental health of older people.

The associations described between functionality and QoL, depression and loneliness emphasize the importance of developing and implementing public policies that simultaneously promote mental and physical health through measures aimed at improving the quality of long-term care. Thus, the results of the present study underscore the need to develop multifaceted approaches to promote functionality and independence in aging and cognitively vulnerable populations.

This study is particularly relevant because it focuses on a vulnerable group and offers a robust basis for formulating more effective and personalized health policies that are aligned with the diverse needs of a growing older population. It also contributes to reducing social and health inequalities, with pioneering impacts in health promotion, human rights and health governance through the promotion of an innovative and equitable society that values the physical and mental health of institutionalized older adults simultaneously.

Future investigations should longitudinally explore the impact of specific interventions on the functionality and psychosocial well-being of institutionalized older people. In addition, comparative studies between different regions of Portugal could enrich the understanding of regional variations in geriatric care needs and responses.

Nursing care is fundamental to ensuring that the physical, emotional and psychosocial needs of the older are met holistically, promoting healthy and dignified aging in any care setting. Nurses can play a key role in improving self-care and functionality through the promotion of health education, health literacy, physical exercise and cognitive training [56]. By addressing both the challenges and opportunities associated with population aging, healthy and dignified aging can be promoted for all older citizens in Portugal.

## Figures and Tables

**Table 1:** Sociodemographic, clinical and psychological characteristics of the participants.

	*n* = 1303
Sex, *n* (%)	
Male	376 (28.9)
Female	927 (71.1)
Age (years)	
Mean (min–max)	86 (65–104)
Marital status, *n* (%)	
Single	146 (11.2)
Married/In a de facto relationship	218 (16.7)
Widower	898 (68.9)
Divorced/Separated	41 (3.2)
Education, *n* (%)	
Did not attend school and cannot read or write	421 (32.3)
Did not attend school, but can read and write	145 (11.1)
Attended school but not higher education	705 (54.1)
Attended higher education	32 (2.5)
Multimorbidity *, *n* (%)	
No	215 (15.9)
Yes	1085 (84.1)
Cognitive impairment (MMSE) ^¥^, *n* (%)	
No	447 (35.3)
Yes	820 (64.7)
Quality of life (WHOQOL-BREF) ^§^, mean (SD) ^¶^	
Physical domain	53.3 (11.2)
Psychological domain	55.2 (13.1)
Domain of social relationships	60.6 (13.9)
Environmental domain	63.8 (13.7)
General quality of life	52.3 (19.2)
Depressive symptoms (PHQ9) ^£^, median (SD) ^¶^	
Overall score	5.1 (5.0)
Loneliness (UCLA-16) ^µ^, median (SD) ^¶^	
Overall score	29.5 (10.2)

* Diagnosis of two or more chronic diseases; ^¥^ Mini Mental State Examination, MMSE (range: 0–30). Cognitive impairment: a score ≤ 15 for individuals with no formal education; a score ≤ 22 for individuals with <12 years of education; and a score ≤ 27 for individuals with 12 years of education or more. ^§^ World Health Organization Quality of Life–BREF, WHOQOL-BREF (range: 0–100); ^£^ 9-Item Patient Health Questionnaire, PHQ-9 (range: 0–27); ^µ^ The Loneliness Scale for Portuguese Elderly, UCLA (range:16–64); ^¶^ *n* = 447.

**Table 2:** Crude association between sociodemographic and clinical characteristics of older adults and functionality.

	Functionality (ENCS) Crude β (95% CI)				
	General	Self-Care	Learning and Mental Functions	Communication	Relationships
Sex					
Female vs. male	**0.27 (0.14 to 0.39)**	**0.33 (0.18 to 0.47)**	**0.31 (0.16 to 0.45)**	**0.17 (0.02 to 0.32)**	0.02 (−0.06 to 0.09)
Age (years)	**0.01 (0.01 to 0.02)**	**0.02 (0.01 to 0.03)**	**0.02 (0.01 to 0.03)**	0.01 (−0.00 to 0.02)	0.00 (−0.00 to 0.01)
Marital status					
Married/In a de facto relationship vs. Single, widowed or divorced/separated	−0.01 (−0.16 to 0.14)	−0.02 (−0.20 to 0.15)	0.01 (−0.17 to 0.19)	−0.05 (−0.24 to 0.13)	0.05 (−0.04 to 0.15)
Education					
Attended school vs. Did not attend school	**−0.45 (−0.56 to −0.34)**	**−0.44 (−0.57 to −0.31)**	**−0.57 (−0.70 to −0.44)**	**−0.48 (−0.62 to −0.35)**	**−0.26 (−0.33 to −0.18)**
Multimorbidity *					
Yes vs. No	0.05 (−0.10 to 0.21)	0.06 (−0.12 to 0.24)	0.06 (−0.12 to 0.25)	0.06 (−0.13 to 0.25)	**−0.10 (−0.20 to −0.01)**
Cognitive impairment (MMSE) ^¥^					
Yes vs. No	**1.24 (1.15 to 1.34)**	**1.28 (1.16 to 1.40)**	**1.53 (1.41 to 1.65)**	**1.38 (1.25 to 1.50)**	**0.36 (0.29 to 0.43)**

Crude β, unstandardized beta; 95% CI, 95% confidence interval. * Diagnosis of two or more chronic diseases; ^¥^ Mini Mental State Examination, MMSE (range: 0–30). Cognitive impairment: a score ≤ 15 for individuals with no formal education; a score ≤ 22 for individuals with <12 years of education; and a score ≤ 27 for individuals with 12 years of education or more. Bold type indicates statistically significant associations (*p* < 0.05).

**Table 3:** Crude and adjusted associations between participants’ sociodemographic, clinical and psychosocial characteristics and severe or complete impaired functioning.

	Functionality (ENCS)	
	Severe or Complete Problem ^1^	
	Crude OR (95% CI)	Adjusted OR (95% CI) ^2^
Sex		
Male	1	-
Female	**1.41 (1.10–1.81)**	-
Age (years)		
<80	1	-
≥80	0.88 (0.65–1.20)	-
Marital status		
Married/In a de facto relationship	1	-
Single, widowed or divorced/separated	0.91 (0.68–1.22)	-
Education (years)		
Did not attend school	1	-
Attended school	**0.46 (0.37–0.58)**	-
Multimorbidity *		
No	1	-
Yes	1.15 (0.84–1.56)	-
Cognitive impairment (MMSE) ^¥^		
No	1	-
Yes	**22.07 (14.60–33.36)**	-
Quality of life (WHOQOL-BREF) ^§^		
Physical domain	**0.96 (0.93–0.99)**	**0.96 (0.93–0.99)**
Psychological domain	0.99 (0.96–1.02)	0.99 (0.96–1.02)
Domain of social relationships	**0.96 (0.93–0.98)**	**0.96 (0.94–0.99)**
Environmental domain	**0.96 (0.93–0.98)**	**0.96 (0.93–0.99)**
Overall quality of life	0.99 (0.97–1.01)	0.99 (0.97–1.01)
Depressive symptoms (PHQ9) ^£^		
None, minimal or mild	1	1
Moderate, moderately severe or severe	**3.28 (1.64–6.55)**	**3.69 (1.80–7.52)**
Negative feelings of loneliness (UCLA-16) ^µ^		
No	1	1
Yes	**2.01 (1.07–3.78)**	**2.04 (1.07–3.87)**

* Diagnosis of two or more chronic diseases; ^¥^ Mini Mental State Examination, MMSE (range: 0–30); Cognitive impairment: a score ≤ 15 for individuals with no formal education; a score ≤ 22 for individuals with <12 years of education; and a score ≤ 27 for individuals with 12 years of education or more. ^§^ World Health Organization Quality of Life–BREF, WHOQOL-BREF (range: 0–100); ^£^ 9-Item Patient Health Questionnaire, PHQ-9: none, minimal or mild depressive symptoms, 0–14 points; moderate, moderately severe or severe depressive symptoms, 15–27 points; ^µ^ Loneliness Scale for Portuguese Elderly, UCLA: feelings of loneliness, >32 points. ^1^ Elderly Nursing Core Set, ENCS: no, mild or moderate problem: 0–49%; severe or complete problem: 50–100%. ^2^ Adjusted for sex, age and education level. Bold type indicates statistically significant associations (*p* < 0.05).

## Data Availability

Data will be available upon request.

## Public Involvement Statement

No public involvement in any aspect of this research.

## Guidelines and Standards Statement

This manuscript was drafted against the STROBE (The Strengthening the Reporting of Observational Studies in Epidemiology Statement) for observational research.

## References

[1] Jones C.H., Dolsten M. (2024). Healthcare on the brink: Navigating the challenges of an aging society in the United States. NPJ Aging.

[2] United Nations Department of Economic and Social Affairs (UNDESA) (2019). World Population Aging 2019: Highlights.

[3] World Health Organization (WHO) (2015). World Report on Aging and Health.

[4] United Nations Population Fund (UNFPA) (2023). 8 Billion Lives, Infinite Possibilities: In Defense of Rights and Choices. World Population Status Report 2023..

[5] Chen H.L., Yu X.H., Yin Y.H., Shan E.F., Xing Y., Min M., Ding Y.P., Fei Y., Li X.W. (2023). Multimorbidity patterns and the association with health status of the oldest-old in long-term care facilities in China: A two-step analysis. BMC Geriatr..

[6] Maresova P., Javanmardi E., Barakovic S., Barakovic Husic J., Tomsone S., Krejcar O., Kuca K. (2019). Consequences of chronic diseases and other limitations associated with old age: A scoping review. BMC Public Health.

[7] Bôas S.S.V., de Araújo C.M., Prates R.V., Novais M.M., Pinto D.S., dos Reis L.A. (2020). Functional capacity and family support in long-lived elderly people living at home. Saúde.

[8] Cochar-Soares N., Delinocente M.L., Dati L.M. (2021). Physiology of aging: From plasticity to cognitive consequences. Rev. Neurocienc..

[9] Denkinger M., Knol W., Cherubini A., Simonds A., Lionis C., Lacombe D., Petelos E., McCarthy M., Ouvrard P., Van Kerrebroeck P. (2023). Inclusion of functional measures and frailty in the development and evaluation of medicines for older adults. Lancet Healthy Longev..

[10] Menassa M., Stronks K., Khatmi F., Roa Díaz Z.M., Espinola O.P., Gamba M., Itodo O.A., Buttia C., Wehrli F., Minder B. (2023). Concepts and definitions of healthy ageing: A systematic review and synthesis of theoretical models. EClinicalMedicine.

[11] Fonseca C., Pinho L.G., Lopes M.J., Marques M.C., Garcia-Alonso J. (2021). The Elderly Nursing Core Set and the cognition of Portuguese older adults: A cross-sectional study. BMC Nurs..

[12] Lee R.Z.Y., Yang W.F.Z., Mahendran R., Suárez L. (2024). Psychometric properties of the World Health Organization WHOQOL-AGE Scale in Singapore. Eur. J. Ageing.

[13] Imaginário C., Rocha M., Machado P., Antunes C., Martins T. (2020). Functional capacity and self-care profiles of older people in senior care homes. Scand. J. Caring Sci..

[14] Lopes M., Sakellarides C. (2021). Os Cuidados de Saúde Face aos Desafios do Nosso Tempo-Contributos para a Gestão da Mudança.

[15] Schmidt A.M., Laurberg T.B., Moll L.T., Schiøttz-Christensen B., Maribo T. (2020). The effect of an integrated multidisciplinary rehabilitation programme for patients with chronic low back pain: Long-term follow up of a randomized controlled trial. Clin. Rehabil..

[16] Jerez-Roig J., de Brito Macedo Ferreira L.M., Torres de Araújo J.R., Costa Lima K. (2017). Functional decline in nursing home residents: A prognostic study. PLoS ONE.

[17] Birtwell K., Planner C., Hodkinson A., Hall A., Giles S., Campbell S., Tyler N., Panagioti M., Daker-White G. (2022). Transitional Care Interventions for Older Residents of Long-term Care Facilities A Systematic Review and Meta-analysis. JAMA Netw. Open.

[18] Gomes G., Moreira R., Maia T., dos Santos M.A., Silva V. (2021). Factors associated with personal autonomy among the elderly: A systematic review of the literature. Ciência Saúde Coletiva.

[19] Geigl C., Loss J., Leitzmann M., Janssen C. (2023). Social factors of health-related quality of life in older adults: A multivariable analysis. Qual. Life Res..

[20] Pinho L.G., Lopes M.J., Correia T., Sampaio F., Arco H.R., Mendes A., Marques M.C., Fonseca C. (2021). Patient-Centered Care for Patients with Depression or Anxiety Disorder: An Integrative Review. J. Pers. Med..

[21] Tomás M.T., Galán-Mercant A., Carnero E.A., Fernandes B. (2018). Functional Capacity and Levels of Physical Activity in Aging: A 3-Year Follow-up. Front. Med..

[22] Nguyen T.X., Nguyen A.H.P., Nguyen H.T.T., Nguyen T.T.H., Nguyen H.L., Goldberg R.J., Thillainadesan J., Naganathan V., Vu H.T.T., Tran L.V. (2024). Health-Related Quality of Life among Older Adults with Dementia Living in Vietnamese Nursing Homes. Int. J. Environ. Res. Public Health.

[23] Bäuerle A., Steinbach J., Schweda A., Beckord J., Hetkamp M., Weismüller B., Kohler H., Musche V., Dörrie N., Teufel M. (2020). Mental health burden of the COVID-19 outbreak in Germany: Predictors of mental health impairment. J. Prim. Care Community Health.

[24] Hwang T.J., Rabheru K., Peisah C., Reichman W., Ikeda M. (2020). Loneliness and social isolation during the COVID-19 pandemic. Int. Psychogeriatr..

[25] Ratcliffe J., Galdas P., Kanaan M. (2024). Older men and loneliness: A cross-sectional study of sex differences in the English Longitudinal Study of Aging. BMC Public Health.

[26] PORDATA (2023). [Aging Index and Other Aging Indicators]. PORDATA. https://www.pordata.pt/portugal/indice+de+envelhecimento+e+outros+indicadores+de+envelhecimento-526.

[27] Flaubert J.L., Le Menestrel S., Williams D.R., Wakefield M.K., National Academies of Sciences, Engineering, and Medicine, National Academy of Medicine, Committee on the Future of Nursing 2020–2030 (2021). The Role of Nurses in Improving Health Care Access and Quality. The Future of Nursing 2020–2030: Charting a Path to Achieve Health Equity.

[28] Fonseca C., Christian K. (2018). The Dimension of sustainable indicators for rehabilitation nursing care and its implications for people aged 65 and over with self-care deficit: Systematic Review of Literature. J. Aging Innov..

[29] Muszalik M., Repka I., Puto G., Kowal-Skałka J., Kędziora-Kornatowska K. (2021). Assessment of Functional Status and Quality of Life of Elderly Patients Undergoing Radiotherapy and Radiotherapy Combined with Chemotherapy—A Cross-Sectional Study. Clin. Interv. Aging.

[30] Folstein M.F., Folstein S.E., McHugh P.R. (1975). Mini-mental state: A practical method for grading the cognitive state of patients for the clinician. J. Psychiatr. Res..

[31] Santana I., Duro D., Lemos R., Costa V., Pereira M., Simões M.R., Freitas S. (2016). Mini-mental state examination: Assessment of new normative data in the screening and diagnosis of cognitive impairment. Acta Med. Port..

[32] Vaz-Serra A., Canavarro M.C., Simões M.R., Pereira M., Gameiro S., Quartilho M., Rijo D., Carona C., Paredes T. (2006). Psychometric studies of the World Health Organization quality of life assessment instrument (WHOQOL-Bref) for Portuguese from Portugal. Psiquiatr. Clín..

[33] Canavarro M.C., Simões M.R., Vaz Serra A., Pereira M., Rijo D., Quartilho M.J., Carona C., Simões M., Machado C., Gonçalves M., Almeida L. (2007). Instrumento de avaliação da qualidade de vida da Organização Mundial de Saúde: WHOQOL-Bref. Avaliação Psicológica: Instrumentos Validados para a População Portuguesa.

[34] Monteiro S., Torres A., Pereira A., Albuquerque E., Morgadinho R. (2013). 2077—Preliminary validation study of a portuguese version of the patient health questionnaire (PHQ-9). Eur. Psychiatry.

[35] Ferreira N., Sousa I., Salgado J. (2019). Brief assessment of depression: Psychometric properties of the Portuguese version of the Patient Health Questionnaire (PHQ-9). Psychol. Pract. Res. J..

[36] Russell D. (1996). UCLA Loneliness Scale (Version 3): Reliability, Validity, and Factor Structure. J. Pers. Assess..

[37] Pocinho M., Farate C., Dias C.A., Validação Psicométrica da Escala UCLA-Loneliness para Idosos Portugueses Interações Soc. E Novas Mod. 2010, 10..

[38] Wang J., Xiao L.D., Wang K., Luo Y., Li X. (2020). Gender Differences in Cognitive Impairment among Rural Elderly in China. Int. J. Environ. Res. Public Health.

[39] Lohman M., Dumenci L., Mezuk B. (2020). Gender differences in the construct overlap of frailty and depression: Evidence from the Health and Retirement Study. J. Am. Geriatr. Soc..

[40] Lyu J., Kim H.Y., Han J.W., Kim T.H., Kim K.W. (2021). Cognitive impairment, depression, comorbidity, and disability as determinants of health-related quality of life in older adults. Geriatr. Gerontol. Int..

[41] Plasencia G., Gray S.C., Hall I.J., Smith J.L. (2024). Multimorbidity clusters in adults 50 years or older with and without a history of cancer: National Health Interview Survey, 2018. BMC Geriatr..

[42] Whitty C.J.M., MacEwen C., Goddard A., Alderson D., Marshall M., Calderwood C., Atherton F., McBride M., Atherton J., Stokes-Lampard H. (2020). Rising to the challenge of multimorbidity. BMJ.

[43] Zaninotto P., Batty G.D., Stenholm S., Kawachi I., Hyde M., Goldberg M., Westerlund H., Vahtera J., Head J. (2020). Socioeconomic Inequalities in Disability-free Life Expectancy in Older People from England and the United States: A Cross-national Population-Based Study. J. Gerontol. A Biol. Sci. Med. Sci..

[44] Mendes L., Oliveira J., Barbosa F., Castelo-Branco M. (2022). A Conceptual View of Cognitive Intervention in Older Adults with and Without Cognitive Decline-A Systemic Review. Front. Aging.

[45] Zhang X., Li J., Xie F., Chen X., Xu W., Hudson N.W. (2022). The relationship between adult attachment and mental health: A meta-analysis. J. Pers. Soc. Psychol..

[46] Park S., Kim Y., Yoon S., Nam Y.J., Hong S., Cho Y.H., Son S.J., Hong C.H., Noh J.S., Roh H.W. (2024). Association of Geriatric Depressive Symptoms and Government-Initiated Senior Employment Program: A Population-Based Study. Psychiatry Investig..

[47] Ribeiro O., Teixeira L., Araújo L., Rodríguez-Blázquez C., Calderón-Larrañaga A., Forjaz M.J. (2020). Anxiety, Depression and Quality of Life in Older Adults: Trajectories of Influence across Age. Int. J. Environ. Res. Public Health.

[48] Zhang D., Zheng W., Li K. (2024). A relação entre o estado civil e o comprometimento cognitivo em idosos chineses: Os múltiplos efeitos mediadores do apoio social e da depressão. BMC Geriatr..

[49] Instituto Nacional de Estatística (INE) (2023). Annual Resident Population Estimates.

[50] Davidson P.M., DiGiacomo M., McGrath S.J. (2011). The Feminization of Aging: How Will This Impact on Health Outcomes and Services?. Health Care Women Int..

[51] Smith S.C., Fisher W.P., Cano S.J. (2023). Measuring Health-Related Quality of Life in Dementia. Person-Centered Outcome Metrology.

[52] Chang J., Chen H., Wang Y. (2021). Gender differences in disability among older adults in China: Evidence from the China Health and Retirement Longitudinal Study. J. Aging Health.

[53] Smith M.L., Ory M.G., Ahn S., Kulinski K.P., Jiang L., Horel S. (2020). Regional variations in functional disability and health status among older adults with arthritis: Implications for health disparities. J. Aging Health.

[54] Verropoulou G., Tsimbos C., Papadopoulos A. (2021). Gender disparities in health and functional status among older adults in the Mediterranean region: The role of sociodemographic factors. Eur. J. Aging.

[55] Bucholc M., McClean S.I., Cleland J. (2021). Socioeconomic inequalities in health and disability in older adults: Evidence from the UK Biobank study. J. Epidemiol. Community Health.

[56] Alves E., Gonçalves C., Oliveira H., Ribeiro R., Fonseca C. (2024). Health-related outcomes of structured home-based rehabilitation programs among older adults: A systematic literature review. Heliyon.

